# Operationalization of a frailty index among older adults in the InCHIANTI study: predictive ability for all-cause and cardiovascular disease mortality

**DOI:** 10.1007/s40520-020-01478-3

**Published:** 2020-01-31

**Authors:** Emiel O. Hoogendijk, Sari Stenholm, Luigi Ferrucci, Stefania Bandinelli, Marco Inzitari, Matteo Cesari

**Affiliations:** 1 Department of Epidemiology and Biostatistics, Amsterdam Public Health Research Institute, Amsterdam UMC - Location VU University Medical Center, Amsterdam, The Netherlands; 2 Department of Public Health, University of Turku and Turku University Hospital, Turku, Finland; 3 Centre for Population Health Research, University of Turku and Turku University Hospital, Turku, Finland; 4 National Institute on Aging, Baltimore, MD USA; 5 Geriatric Unit, Azienda Sanitaria di Firenze, Florence, Italy; 6 REFiT Research Group, Vall D’Hebrón Institute of Research and Parc Sanitari Pere Virgili, Barcelona, Spain; 7 Department of Medicine, Universitat Autònoma de Barcelona, Bellaterra, Spain; 8 Geriatric Unit, Fondazione IRCCS Ca’ Granda Ospedale Maggiore Policlinico, Milan, Italy; 9 Department of Clinical Sciences and Community Health, University of Milan, Milan, Italy

**Keywords:** Frail elderly, Frailty index, Deficit accumulation, Risk prediction, Geriatrics

## Abstract

**Background:**

The frailty index (FI) is a sensitive instrument to measure the degree of frailty in older adults, and is increasingly used in cohort studies on aging.

**Aims:**

To operationalize an FI among older adults in the “Invecchiare in Chianti” (InCHIANTI) study, and to validate its predictive capacity for mortality.

**Methods:**

Longitudinal data were used from 1129 InCHIANTI participants aged ≥ 65 years. A 42-item FI was operationalized following a standard procedure using baseline data (1998/2000). Associations of the FI with 3- and 6-year all-cause and cardiovascular disease (CVD) mortality were studied using Cox regression. Predictive accuracy was estimated by the area under the ROC curve (AUC), for a continuous FI score and for different cut-points.

**Results:**

The median FI was 0.13 (IQR 0.08–0.21). Scores were higher in women, and at advanced age. The FI was associated with 3- and 6-year all-cause and CVD mortality (HR range per 0.01 FI increase = 1.03–1.07, all *p* < 0.001). The continuous FI score predicted the mortality outcomes with moderate-to-good accuracy (AUC range 0.72–0.83). When applying FI cut-offs between 0.15 and 0.35, the accuracy of this FI for predicting mortality was moderate (AUC range 0.61–0.76). Overall, the predictive accuracy of the FI was higher in women than in men.

**Conclusions:**

The FI operationalized in the InCHIANTI study is a good instrument to grade the risk of all-cause mortality and CVD mortality. More measurement properties, such as the responsiveness of this FI when used as outcome measure, should be investigated in future research.

**Electronic supplementary material:**

The online version of this article (10.1007/s40520-020-01478-3) contains supplementary material, which is available to authorized users.

## Introduction

In aging societies, there is increasing attention for the concept of frailty [1, 2]. This is an age-related condition, which has been defined as a decline in multiple physiological systems and increased vulnerability to stressors. Frailty is strongly related to various adverse health outcomes, such as functional decline, delirium, hospital admission, and mortality [3].

In the past decades, many frailty instruments have been developed [4], of which the frailty index (FI) is one of the most commonly used [5]. The FI defines frailty as a gradient from fit to frail, based on the accumulation of health deficits [5]. A critical mass of health deficits (at least 30), including diseases, disabilities, signs, and symptoms, is used to arithmetically generate a continuous score ranging from 0 to 1. As a result, the FI seems to be a sensitive instrument to distinguish the degree of frailty in older adults [6].

Because of its sensitivity, the FI is increasingly recognized as particularly useful for longitudinal studies on aging, as predictor of adverse health events or as outcome measure. As an outcome measure, the FI can be used to monitor the individual`s changes in frailty over time [7, 8]. Recently, FIs have been developed in various longitudinal studies on aging, such as the English Longitudinal Study of Aging (ELSA) [9], the Longitudinal Aging Study Amsterdam (LASA) [10, 11], the Health and Retirement Study (HRS) [12], the Survey of Health, Aging and Retirement in Europe (SHARE) [13, 14], and the Gothenburg H70 Birth Cohort Studies (H70 Studies) [15]. This is a promising development, as this enables comparisons between different populations, countries, and settings.

The FI was originally developed to be used as a continuous scale [16]. However, dichotomizing a measure is sometimes necessary to be able to identify the presence of certain conditions and to inform clinicians in the development of diagnostic/therapeutic plans. Although cut-offs are controversial, they have also been applied to the FI to determine the presence of frailty. Some studies have reported an FI cut-off of 0.2 to identify a frail state [17], but a cut-point of 0.25 has most often been used (e.g., [16, 18–20]). Interestingly, there is a lack of validation studies for these cut-offs with regard to outcome prediction. Therefore, optimal FI cut-offs for predicting specific adverse health outcomes are largely unknown. Furthermore, most FIs have been validated for all-cause mortality (e.g., [11, 13]. Very few studies have investigated the predictive validity for specific causes of death, such as cardiovascular disease (CVD) mortality [21].

The “Invecchiare in Chianti”, (i.e., Aging in the Chianti area; InCHIANTI) study is a population-based cohort study of older adults in Tuscany, Italy. The study is well known for its contribution to the frailty research field, especially with regard to insights into determinants and onset of physical frailty in later life [22–28]. Nevertheless, all previous InCHIANTI studies measured frailty using the frailty phenotype [29]. An FI has not yet been established in this cohort study. Therefore, our aim was to operationalize an FI in the InCHIANTI study, and to validate it by investigating its predictive ability for all-cause mortality and CVD mortality. Both continuous scores and different cut-offs were used to study the predictive accuracy of this FI.

## Methods

### Study sample

The InCHIANTI study is a prospective population-based cohort study among older adults aged 65 years and over in Tuscany, Italy. Participants were randomly selected from population registers in this area. The study started in 1998 and is still ongoing. Follow-up measurements are conducted every 3 years. The main focus of InCHIANTI is mobility decline in later life and related factors. The data collection is based on a home interview and clinical measurements at the study clinic. Details on the sampling and design of InCHIANTI have been described in a previous publication [30]. The InCHIANTI study was approved by the ethical committee of the Italian National Institute of Research and Care on Aging. All study participants provided written informed consent.

In the current study, we included people with valid data at baseline (1998–2000) and valid data on the outcome measure (mortality) during follow-up. Of the 1155 InCHIANTI participants at baseline, 24 (2.1%) had no valid frailty score due to missing data and 2 (0.2%) had no data on level of education. Vital status at follow-up was available for all participants, resulting in a sample of 1129 people that were included in the analyses.

### Frailty index

An FI of health deficits was operationalized following a standard procedure, as described by Searle et al. [17]. In the current study, this was done with data from the InCHIANTI baseline. However, we made sure that the included variables are available at all InCHIANTI follow-up waves, so that future longitudinal studies may use this FI as well. Variables were included in the FI if they met various criteria: (a) variables must be health-related deficits, such as symptoms, signs, diseases, or disabilities, all associated with adverse outcomes (b) the deficits are biologically meaningful and represent multiple organ systems, (c) deficits increase with age, but do not saturate too early (i.e., high prevalence at young age), and (d) variables contain less than 5% missing values.

The questionnaires and clinical measurements of InCHIANTI were screened for variables that could be included in the FI. This resulted in a list of health deficits from various functional domains that were used to construct a 42-item FI. The FI included the following variables: major medical conditions (hypertension, myocardial infarction, congestive heart failure, chronic liver disease, cancer, peripheral arterial disease, stroke, Parkinson`s disease, diabetes, chronic lung disease, angina pectoris, and knee/hip arthritis) based on disease ascertainment algorithms (including information from medical records, medication use, signs and symptoms, medical history, and hospital records); having difficulties with various (instrumental) activities of daily living (ADL/IADL, including bathing, dressing/undressing, eating, toileting, continence, walking across a small room, walking up/down staircase ten steps, getting in/out of bed, food preparation, shopping, heavy housework, using telephone, lifting/carry shopping bag, using public transportation, medication use, and managing finances); self-rated health assessed with the question “How would you evaluate your current health?”; five items from the Center for Epidemiologic Studies Depression (CES-D) scale (feel depressed, feel everything is an effort, could not get going, feel lonely, and feel happy) [31]; four sub-domains of the Mini-Mental State Examination (MMSE, domains: orientation time, orientation place, attention, and recall) [32]; self-reported weight loss in the last 12 months; physical activity level in the last year; and two physical performance measures (gait speed, grip strength). Details of the items included in the FI and their cut-offs are shown in Table 1.

**Table 1:** Frailty index items and coding

No.	Deficit	Cut-off
1	Hypertension	No = 0, possible = 0.5, yes = 1
2	Myocardial infarction	No = 0, possible = 0.5, yes = 1
3	Congestive heart failure	No = 0, possible = 0.5, yes = 1
4	Chronic liver disease	No = 0, possible = 0.5, yes = 1
5	Cancer	No = 0, yes = 1
6	Peripheral arterial disease	No = 0, possible = 0.5, yes = 1
7	Stroke	No = 0, possible/TIA = 0.5, yes = 1
8	Parkinson`s disease	No = 0, possible = 0.5, yes = 1
9	Diabetes	No = 0, possible = 0.5, yes = 1
10	Chronic lung disease	No = 0, possible = 0.5, yes = 1
11	Angina pectoris	No = 0, possible = 0.5, yes = 1
12	Knee/hip arthritis	No = 0, pain or stiffness = 0.5, pain and stiffness = 1
13	Bathing	All these ADL/IADL items were coded as: no difficulty = 0 with difficulty but without help = 0.33 with some help from another person = 0.66 unable to do it = 1
14	Dressing/undressing	
15	Eating	
16	Toileting	
17	Continence	
18	Walking across small room	
19	Walking up/down staircase ten steps	
20	Getting in/out of bed	
21	Food preparation	
22	Shopping	
23	Heavy housework	
24	Using telephone	
25	Lifting/carry shopping bag	
26	Using public transportation	
27	Medication use	
28	Managing finances	
29	Self-rated health	Very good = 0, good = 0.25, fair = 0.50, poor = 0.75, very poor = 1
30	Feel depressed (CES-D)	All these CES-D items were coded as: rarely or never = 0 sometimes = 0.33 occasionally = 0.66 often or always = 1
31	Feel everything is an effort (CES-D)	
32	Could not get going (CES-D)	
33	Feel lonely (CES-D)	
34	Feel happy (CES-D)	Often or always = 0, occasionally = 0.33, sometimes = 0.66, rarely or never = 1
35	Orientation time (MMSE)	Five correct = 0, one wrong = 0.50, two or more wrong = 1
36	Orientation place (MMSE)	Five correct = 0, one wrong = 0.50, two or more wrong = 1
37	Attention (MMSE)	Five correct = 0, one or two wrong = 0.50, three or more wrong = 1
38	Recall (MMSE)	Three correct = 0, two correct = 0.50, one or zero correct = 1
39	Weight loss	No = 0, yes, (weight loss > 10 lbs. in past year) = 1
40	Low physical activity	No = 0, yes (hardly any physical activity or < 1 h a week) = 1
41	Slow gait speed	Normal = 0, lowest quintile, stratified by height and sex = 1
42	Low grip strength	Normal = 0, lowest quintile, stratified by BMI and sex = 1

In line with previous studies, we only calculated an FI for participants with less than 20% missing variables [11, 33]. Most older adults in the initial InCHIANTI sample had no missing variables on the FI (65.4%) or 1–3 missing variables (27.2%) out of the total of 42 variables. Only 2.1% had more than 20% missing variables. An FI ranging from 0 (no deficits present) to 1 (all deficits present) was calculated for individual participants by dividing the sum of the items present out of the sum of all the possible ones measured in the FI. To illustrate, if a person presents with 10 altered items out of 42, the corresponding FI score is 10/42 = 0.24.

### Outcomes

Mortality is one of the most studied endpoints in the context of frailty [34]. In the current study, outcome measures were 3-year and 6-year all-cause mortality and CVD mortality. Vital status, date of death, and cause of death (International Classification of Disease, 9^th^ revision (ICD-9) codes) were retrieved from regional and municipality registers. CVD mortality was determined by ICD-9 codes 390–459.

### Statistical analysis

Descriptive analyses were conducted to show the characteristics of the study sample. T tests, Chi-square tests, and Mann–Whitney tests were performed to determine differences in baseline characteristics by sex. Next, descriptive statistics were generated to provide insight into the general characteristics of the FI at baseline. The distribution of the FI was displayed in a histogram. Mean frailty scores were plotted by age and sex. To investigate the predictive ability of the FI, various analyses were performed. First, associations of the FI (continuous score) with each outcome (3-year and 6-year all-cause mortality and CVD mortality) were studied using Cox proportional hazard models, without and with adjustment for confounders. Three models were tested: a crude model (Model 1), a model adjusted for age and sex (Model 2), and a model additionally adjusted for partner status, educational level, and smoking (Model 3). In the Cox regression analyses, survivors were censored at the end of follow-up (3 or 6 years after baseline). People who died within 3 or 6 years were censored at the time of death. In the analyses on CVD mortality, those who died because of other reasons than CVD were also censored at time of death. Second, the predictive accuracy of the continuous FI score was estimated by the area under the ROC curve (AUC). An AUC of 1.0 indicates perfect sensitivity and specificity for the outcome of interest, 0.9–0.99 is excellent, 0.8–0.89 is good, 0.7–0.79 is moderate, and everything below 0.70 is poor [35]. Next, to find optimal FI cut-offs for predicting mortality, we calculated the sensitivity, the specificity, and AUCs for various cut-points (between 0.15 and 0.35) around the commonly used cut-point of 0.25 [16, 18]. All analyses on predictive ability of the FI for all-cause mortality were done for the total population and stratified by sex, as sex differences in frailty have often been reported, with higher frailty levels among women [36]. For CVD mortality, the number of events was too low to perform sex-stratified analyses. All analyses were done in SPSS 24 (IBM corp, Armonk, NY, USA).

## Results

**Fig. 1:**
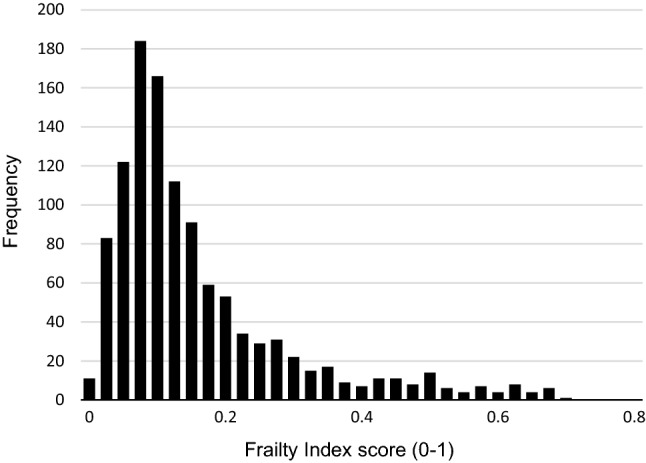
Distribution of the frailty index at baseline

**Fig. 2:**
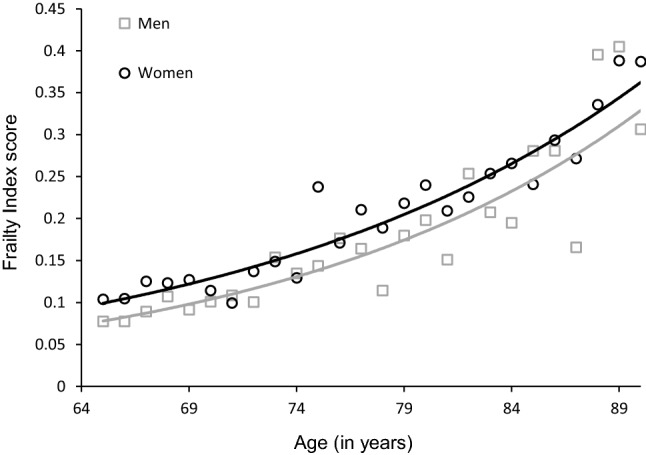
Association between age and baseline frailty index score for men and women^a^. ^a^The circles and squares indicate frailty index scores by age rounded to nearest whole number, and may represent more than one individual

Baseline characteristics are shown in Table 2. The analytical sample consisted of 1129 older adults, of which 642 (56.9%) were female. The participants had a mean age of 75.2 years (SD = 7.4) and a mean educational level of 5.3 years (SD = 3.3). The distribution of the FI is displayed in Fig. 1. The FI is skewed to the right and ranges from 0.01 to 0.72. The median FI score was 0.13 (IQR = 0.08–0.21) and the 99% upper limit was 0.64. When applying cut-offs, the prevalence of frailty ranged from 10.4% (≥ 0.35 cut-off) to 39.9% (≥ 0.15 cut-off). Frailty scores were higher in women than in men (median = 0.14 vs. median = 0.10, *p* < 0.001). Figure 2 shows that the FI scores tend to be higher with advancing age in both men and women.

**Table 2:** Baseline characteristics

Characteristics	Total	Men	Women	*p* value^a^
	*n* = 1129	*n* = 487	*n* = 642	
Age, mean (SD)	75.2 (7.4)	74.2 (7.0)	75.9 (7.7)	< 0.001
Partner status, *n* (%) with partner	674 (59.7)	384 (78.9)	290 (45.2)	< 0.001
Educational level, years, mean (SD)	5.3 (3.3)	6.2 (3.6)	4.7 (2.9)	< 0.001
Smoking, *n* (%) current smoker	158 (14.0)	103 (21.1)	55 (8.6)	< 0.001
ADL disabilities, 0–6, mean (SD)	0.2 (1.0)	0.2 (0.9)	0.3 (1.0)	0.21
CES-D score, 0–60, mean (SD)	13.1 (8.8)	9.9 (7.3)	15.6 (9.0)	< 0.001
MMSE score, 0–30, mean (SD)	24.3 (4.9)	25.2 (4.4)	23.7 (5.2)	< 0.001
FI score, 0–1, median (IQR)	0.13 (0.08–0.21)	0.10 (0.07–0.17)	0.14 (0.10–0.24)	< 0.001
FI cut-offs, *n* (%)				
≥ 0.15	451 (39.9)	144 (29.6)	307 (47.8)	< 0.001
≥ 0.20	301 (26.7)	93 (19.1)	208 (32.4)	< 0.001
≥ 0.25	214 (19.0)	62 (12.7)	152 (23.7)	< 0.001
≥ 0.30	154 (13.6)	47 (9.7)	107 (16.7)	< 0.01
≥ 0.35	117 (10.4)	40 (8.2)	77 (12.0)	< 0.05

^a^Based on t test, Chi-square test, or Mann–Whitney test

**Fig. 3:**
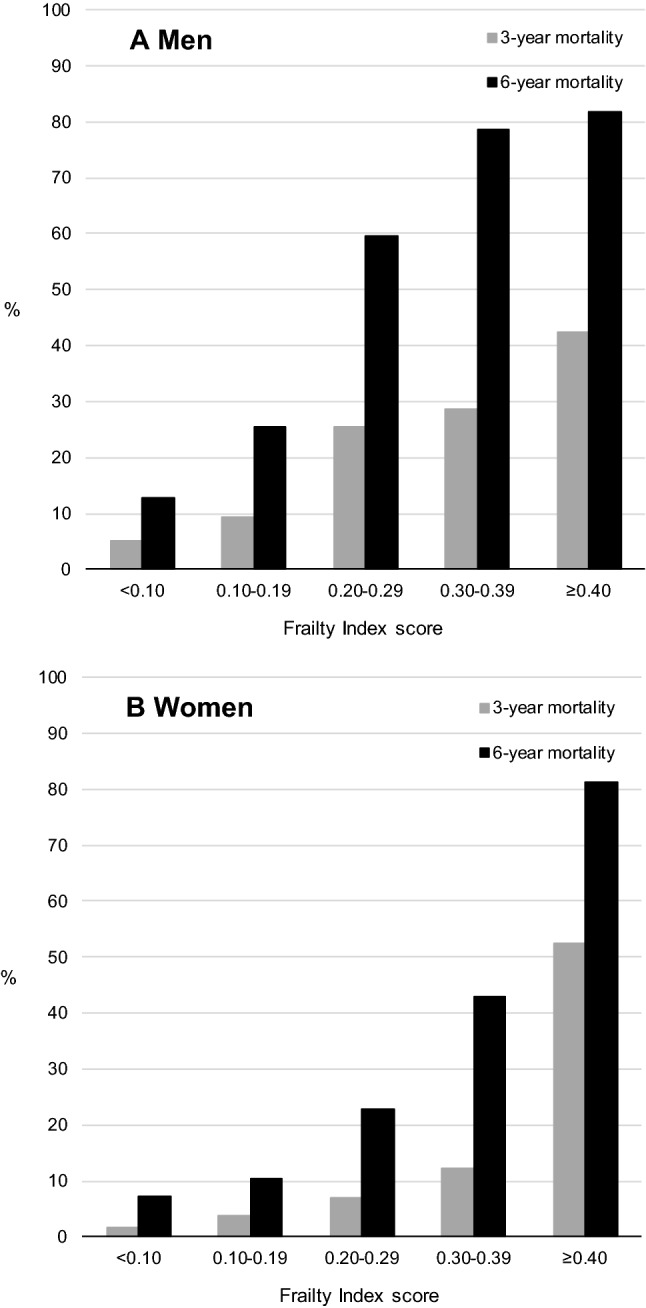
Percentage of all-cause mortality at follow-up by baseline frailty index score for **A** men and **B** women

Table 3 shows the associations of the continuous FI score with all-cause mortality for the total sample as well as after stratification by sex. In the total sample (*n* = 1129), 112 people (9.9%) died within 3 years of follow-up and 267 people (23.6%) died within 6 years of follow-up. Mortality was higher in men than women (3-year mortality = 11.5% vs. 8.7%; 6-year mortality = 27.9% vs. 20.4%, respectively). An increase in all-cause mortality by FI score was observed for both men and women (Fig. 3). Table 3 also shows CVD mortality for the total population. During 3 years and 6 years of follow-up, 53 (4.7%) and 128 people (11.3%) died because of CVD, respectively. The FI showed statistically significant associations with all outcomes in all models. For example, 0.01 increment in the FI score was associated with 3-year all-cause mortality (HR 1.04, 95% CI 1.03–1.06) and 3-year CVD mortality (HR 1.05, 95% CI 1.03–1.06) in models adjusted for age, sex, partner status, educational level, and smoking. The associations between the FI score and all-cause mortality were stronger among women than among men (*p* interaction FI*sex was < 0.05, not shown in table) for both 3-year and 6-year mortality.

**Table 3:** Cox regression: associations between frailty index score and mortality

	All-cause mortality						CVD mortality	
	3 years			6 years			3 years	6 years
	Total	Men	Women	Total	Men	Women	Total	Total
*n* events/total *n*	112/1129	56/487	56/642	267/1129	136/487	131/642	53/1129	128/1129

	HR (95% CI)^a^	HR (95% CI)^a^	HR (95% CI)^a^	HR (95% CI)^a^	HR (95% CI)^a^	HR (95% CI)^a^	HR (95% CI)^a^	HR (95% CI)^a^
Model 1^b^	1.06 (1.05–1.07)	1.05 (1.04–1.06)	1.07 (1.06–1.09)	1.06 (1.05–1.07)	1.05 (1.04–1.06)	1.07 (1.06–1.08)	1.07 (1.05–1.08)	1.07 (1.06–1.08)
Model 2^b^	1.04 (1.03–1.05)	1.03 (1.02–1.05)	1.05 (1.04–1.07)	1.04 (1.03–1.05)	1.03 (1.02–1.04)	1.05 (1.04–1.06)	1.05 (1.03–1.06)	1.04 (1.03–1.06)
Model 3^b^	1.04 (1.03–1.06)	1.03 (1.02–1.05)	1.05 (1.04–1.07)	1.04 (1.03–1.05)	1.03 (1.02–1.05)	1.05 (1.04–1.06)	1.05 (1.03–1.06)	1.04 (1.03–1.06)

*HR* hazard ratio, *95% CI* 95% confidence interval, *CVD* cardiovascular disease

^a^The hazard ratios indicate change in mortality with an increase of 0.01 on the frailty index

^b^Model 1: unadjusted; Model 2: adjusted for age and sex; Model 3: adjusted for age, sex, partner status, educational level, and smoking; Sex adjustment only in the analysis of the total population

The predictive accuracy of the continuous FI score for the studied outcomes (Tables 4 and 5) ranged from moderate to good (AUC range 0.72–0.83), and was a bit higher among women (AUC range 0.80–0.83) compared to men (AUC range 0.72–0.77). For the total population, the AUC was the same for predicting 3-year and 6-year all-cause mortality (AUC 0.76), and nearly the same for predicting 3-year and 6-year CVD mortality (AUC 0.79 vs. AUC 0.78). Tables 4 and 5 also show the predictive accuracy of different FI cut-offs. For prediction of mortality, all cut-offs between 0.15 and 0.35 had moderate accuracy (AUC < 0.80). In the Online Appendix, ROC curves and optimal cut-offs for all outcomes are graphically displayed (see Online Appendix 1). The optimal cut-off for predicting 3-year mortality was 0.198 (AUC 0.71), for 6-year mortality 0.191 (AUC 0.72), for 3-year CVD mortality 0.201 (AUC 0.76), and for 6-year CVD mortality 0.201 (AUC 0.73). Sex differences can be observed with regard to the optimal FI cut-point for outcome prediction. For example, for men, the optimal cut-off for predicting 3-year mortality was around ≥ 0.17, while for women, the optimal cut-off was around ≥ 0.28 (Tables 4 and 5, Online Appendix 1).

**Table 4:** Predictive accuracy of continuous frailty index scores and various frailty index cut-offs for 3-year mortality

	3-Year all-cause mortality									3-Year CVD mortality		
	Total			Men			Women			Total		
	Sens	Spec	AUC (95% CI)	Sens	Spec	AUC (95% CI)	Sens	Spec	AUC (95% CI)	Sens	Spec	AUC (95% CI)
Continuous			0.76 (0.70–0.81)			0.72 (0.64–0.80)			0.83 (0.77–0.89)			0.79 (0.72–0.87)
Cut-off												
≥ 0.15	0.72	0.64	0.68 (0.63–0.73)	0.61	0.74	0.68 (0.60–0.75)	0.84	0.56	0.70 (0.63–0.76)	0.77	0.62	0.70 (0.63–0.77)
≥ 0.20	0.64	0.77	0.71 (0.66–0.76)	0.52	0.85	0.68 (0.60–0.77)	0.77	0.72	0.74 (0.68–0.81)	0.75	0.76	0.76 (0.69–0.83)
≥ 0.25	0.54	0.85	0.70 (0.64–0.75)	0.41	0.91	0.66 (0.57–0.75)	0.68	0.81	0.74 (0.69–0.82)	0.62	0.83	0.73 (0.65–0.81)
≥ 0.30	0.48	0.90	0.69 (0.63–0.75)	0.32	0.93	0.63 (0.54–0.71)	0.64	0.88	0.76 (0.68–0.84)	0.55	0.88	0.72 (0.63–0.80)
≥ 0.35	0.43	0.93	0.68 (0.62–0.74)	0.29	0.94	0.62 (0.53–0.70)	0.57	0.92	0.75 (0.67–0.83)	0.49	0.92	0.70 (0.62–0.79)

*Sens.* Sensitivity, *Spec.* specificity, *AUC* area under the ROC curve, *CVD* cardiovascular disease

**Table 5:** Predictive accuracy of continuous frailty index scores and various frailty index cut-offs for 6-year mortality

	6-Year all-cause mortality									6-Year CVD mortality		
	Total			Men			Women			Total		
	Sens	Spec	AUC (95% CI)	Sens	Spec	AUC (95% CI)	Sens	Spec	AUC (95% CI)	Sens	Spec	AUC (95% CI)
Continuous			0.76 (0.73–0.80)			0.77 (0.72–0.82)			0.80 (0.76–0.85)			0.78 (0.73–0.82)
Cut-off												
≥ 0.15	0.70	0.69	0.69 (0.66–0.73)	0.58	0.81	0.70 (0.64–0.75)	0.82	0.61	0.71 (0.67–0.76)	0.75	0.65	0.70 (0.65–0.75)
≥ 0.20	0.58	0.83	0.71 (0.67–0.75)	0.48	0.92	0.70 (0.64–0.76)	0.69	0.77	0.73 (0.68–0.78)	0.67	0.79	0.73 (0.68–0.78)
≥ 0.25	0.48	0.90	0.69 (0.65–0.73)	0.37	0.97	0.67 (0.61–0.73)	0.60	0.86	0.73 (0.67–0.78)	0.56	0.86	0.71 (0.66–0.77)
≥ 0.30	0.40	0.94	0.67 (0.63–0.71)	0.28	0.97	0.63 (0.57–0.69)	0.52	0.92	0.72 (0.67–0.78)	0.48	0.91	0.69 (0.64–0.75)
≥ 0.35	0.33	0.97	0.65 (0.61–0.69)	0.24	0.98	0.61 (0.55–0.67)	0.42	0.96	0.69 (0.63–0.75)	0.38	0.93	0.65 (0.60–0.71)

*Sens.* sensitivity, *Spec.* specificity, *AUC* area under the ROC curve, *CVD* cardiovascular disease

## Discussion

In the present study, we operationalized a 42-item FI in the InCHIANTI study, a large population-based cohort study. We validated this FI for predicting mortality, and investigated optimal cut-offs for outcome prediction. Our results showed that this FI is associated with 3-year and 6-year all-cause and CVD mortality. It is, therefore, a good instrument to grade the risk of mortality in older adults. The FI predicted mortality with moderate-to-good accuracy, and showed slightly better predictive accuracy in women than in men. Interestingly, the optimal FI cut-off for predicting mortality differed between men and women.

The characteristics of the FI developed in the InCHIANTI study are in line with previously published FIs in older population-based samples. In fact, our FI had a skewed right distribution, a 99% upper limit below 0.70, and higher values in women and persons with more advanced age [11, 17, 37]. The median FI score of 0.13 did not differ much from the FI that was reported in the Longitudinal Aging Study Amsterdam (median = 0.16) in the same time period (late 1990s) [11]. Furthermore, we validated the FI against mortality. Consistent with results from a large number of earlier studies (e.g., [11, 34, 38–40]), a higher FI score in the InCHIANTI study was associated with increased all-cause mortality. A novel finding is that the FI was also related to CVD mortality.

Although sex differences in frailty have been reported in many studies, relatively little attention has been paid to sex differences in the association between frailty and adverse outcomes [36]. A common observation from previous research is that men have higher mortality rates in combination with lower average frailty scores [36]. Our results showed that, even with greater mortality rates among men, a stronger relationship between the FI score and all-cause mortality was observed among women. Perhaps, this association is influenced by the greater increase in mortality in the highest FI categories among women, as can be observed in Fig. 3. These findings suggest that a sex-specific approach to frailty is warranted when the FI is used to predict adverse outcomes. More research is needed to investigate whether sex differences are also present for associations with other outcomes than mortality.

The predictive accuracy of the continuous FI score for mortality was similar to findings from other population-based cohort studies. We observed AUCs of 0.76 for predicting both 3-year and 6-year mortality. This was comparable to the previous work in the SHARE study, which found AUCs for predicting 2-year and 5-year mortality of 0.77 and 0.75, respectively [33]. And it is also not very different from other risk indicators that have been used in older populations, such as gait speed measurement, which also showed moderate predictive accuracy for mortality with AUCs around 0.70 [41]. Nevertheless, it is very likely that the predictive accuracy of the FI differs across settings and subpopulations. For example, in an earlier Italian study, a slightly higher AUC (0.81) for predicting 2-year mortality was observed [38]. The FI was of similar size as ours (40 items), but the main difference is that our study was conducted in a population-based sample, while this previous study was done in a specific sample in a clinical setting. Another study conducted in a clinical setting found; however, moderate predictive accuracy for 1-year, 3-year, and 5-year mortality (AUC ≤ 0.75) when operationalizing a frailty index using data from electronic medical records [39]. This highlights the need for more research into the predictive accuracy of the FI across different settings. Future studies should explore to what extent the predictive ability of the FI in clinical settings differs from that in population-based samples, and whether there is consistency in the predictive ability of the FI within specific settings. It is, for example, possible that the FI is less predictive in long-term care facilities, where average frailty levels are much higher than in other settings [42].

One of the main advantages of using an FI is that the content is not fixed. As long as several conditions are met, such as the type and number of included health deficits (at least 30 items), the combination of health deficits does not matter [17]. The key characteristics of the FI are consistent across data sets with different FI operationalizations [37]. Because of this flexibility, FIs can be constructed with almost any comprehensive health database in both research and clinical settings. The FI that was constructed in the current study contains 42 items from various domains of functioning. However, our aim was not to construct an FI that is completely fixed. In future research with InCHIANTI data, it is still possible to replace items or to add more items, if this is needed for specific research questions.

The FI is not meant to be dichotomized, as Rockwood et al. described in their paper from 2007 [16]. However, when a cut-point is needed, an arbitrary cut-off of ≥ 0.25 has usually been proposed for community-dwelling older adults. Until now, this cut-point has been used in many studies [18–20]. In the present study, we explored the use of various cut-offs around this commonly used cut-point. Whereas the predictive accuracy of the continuous FI was moderate-to-good, the dichotomized scores had slightly lower levels of accuracy. Remarkably, we observed sex differences with regard to the optimal cut-off for outcome prediction. The optimal cut-off for men for predicting 3-year mortality was around 0.17, while for women, this was around 0.28. This could just be an expression of the male–female health-survival paradox (i.e., a higher life expectancy that is accompanied by higher rates of poor health in women compared to men) [36]. It should be noted that for some higher cut-off values, especially among men, the sensitivity was low. It depends on the purpose of the study, but if a dichotomized FI would be used as screening instrument, one would prefer high sensitivity as this would select the highest number of true positive cases (people that experience the adverse health event). Additional research in other cohorts is, therefore, needed, to further investigate optimal FI cut-offs for specific outcomes.

The current study has several strengths. We used data from a large population-based cohort of older adults in Italy to validate an FI that was constructed based on existing FI methodology. Our study expands previous research in various ways. First, this is one the few studies that tests various FI cut-offs for outcome prediction. Second, this study contributes to insights into sex differences in frailty, by providing detailed information on the predictive accuracy of the FI for men and women separately. And, finally, in addition to all-cause mortality, we have also investigated CVD mortality as outcome. Specific causes of death have seldom been studied in relation to the FI [21].

The study also has some limitations. First, we validated the FI only for mortality, an important outcome measure, but other outcomes should be considered in future research, such as falls, functional decline, and healthcare utilization [3]. Second, we only have studied the main characteristics and the predictive accuracy of the FI. More measurement properties need to be investigated. For example, the instrument has great potential to be used as outcome measure. A crucial next step is to test the responsiveness of this FI when used as outcome in longitudinal research. Another important direction for future research is the performance of comparative research, to see to what extent the predictive ability of this FI differs from that of other risk indicators for mortality or other frailty measures.

The application of the FI is not only limited to research settings. It has been suggested that the FI is a good instrument to select older adults in clinical practice that may benefit from additional care or specific interventions [39, 40]. However, this field is in an early stage of development. For example, in the UK, an electronic frailty index (eFI) is currently being implemented for older adults in primary care [39]. Based on generic cut-points of the eFI, the general practitioner has to carry out additional actions for those with moderate-to-severe frailty. Although this is a promising development, it is possible that some refinement and further specificity of cut-points for use in clinical practice are needed in the future. The results of the current study would, for example, implicate that cut-points need to be sex-specific, as men are at risk of mortality at lower FI levels than women.

To conclude, we have operationalized an FI in the InCHIANTI study and validated this FI for predicting mortality. This FI is a good instrument to grade the risk of 3-year and 6-year all-cause and CVD mortality in older adults. The predictive accuracy of the FI was slightly better among women compared to men. Future research should investigate the responsiveness of the FI when using this instrument as outcome measure.

## Electronic supplementary material

Below is the link to the electronic supplementary material.

Supplementary file1 (DOCX 126 kb)
