# Supplementary material for: Operationalization of a frailty index among older adults in the InCHIANTI study: predictive ability for all-cause and cardiovascular disease mortality
Source: Aging Clin Exp Res. 2020 Jan 31;32(6):1025–34. doi: 10.1007/s40520-020-01478-3 (PMC7260260; doi:10.1007/s40520-020-01478-3)
Supplement: Supplementary file 1 — Supplementary file1 (DOCX 126 kb) [file 40520_2020_1478_MOESM1_ESM.docx]

**Appendix 1.** ROC curves for continuous frailty index scores for mortality and optimal cut-offs

**A) Total population: 3-year mortality. Optimal cut-off = 0.1981 (AUC = 0.712)**


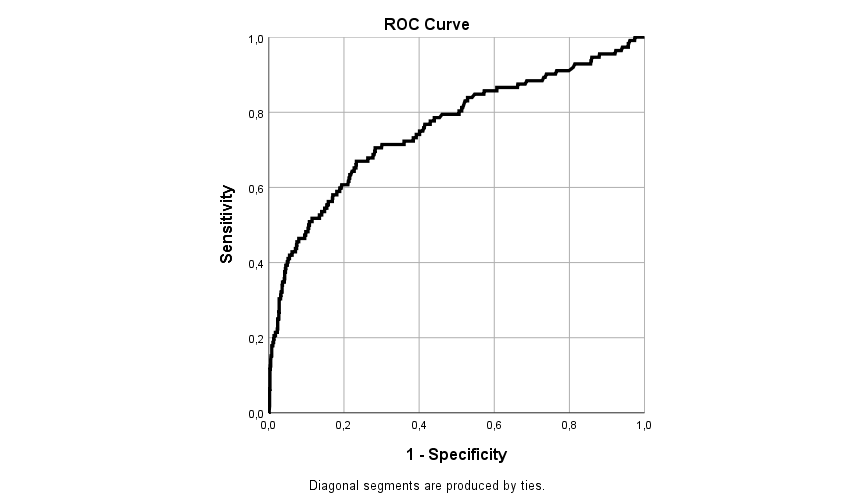


**B) Total population: 6-year mortality. Optimal cut-off = 0.1913 (AUC = 0.715)**


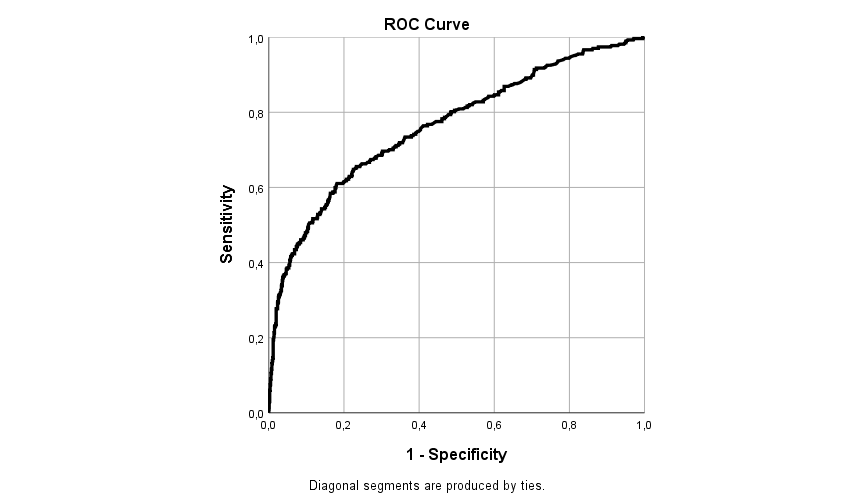


**C) Men: 3-year mortality. Optimal cut-off = 0.1705 (AUC = 0.694)**


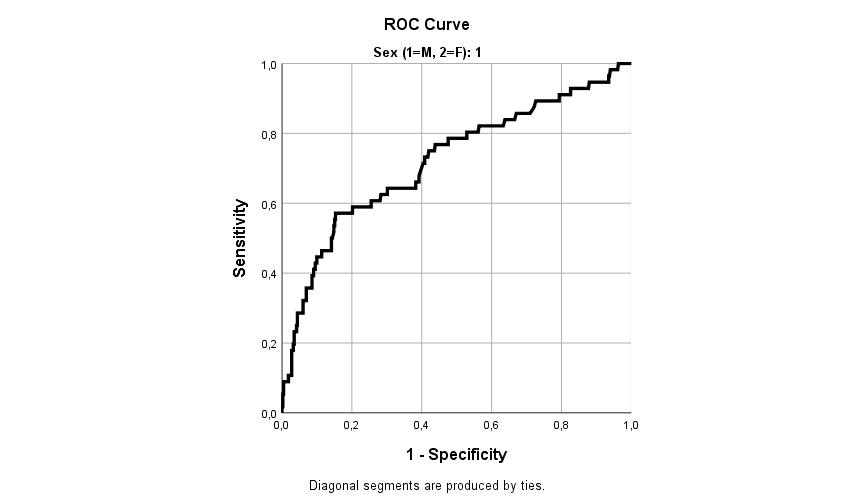


**D) Men: 6-year mortality. Optimal cut-off = 0.1913 (AUC = 0.721)**


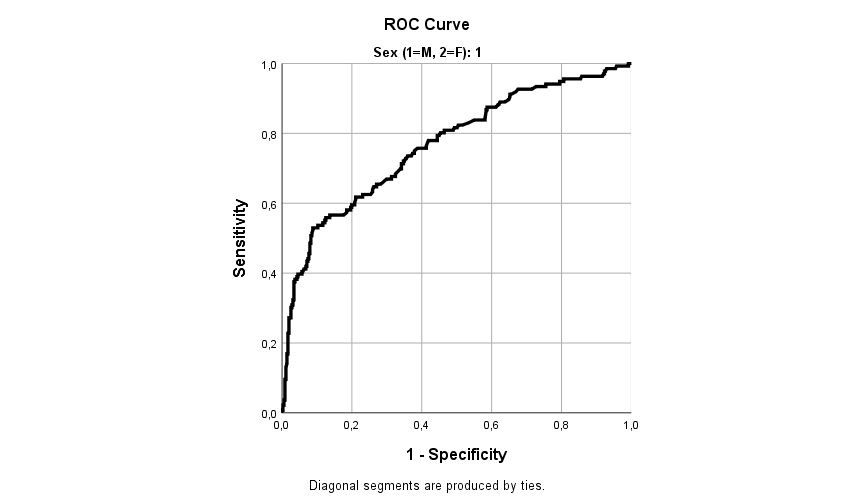


**E) Women: 3-year mortality. Optimal cut-off = 0.2824 (AUC = 0.767)**


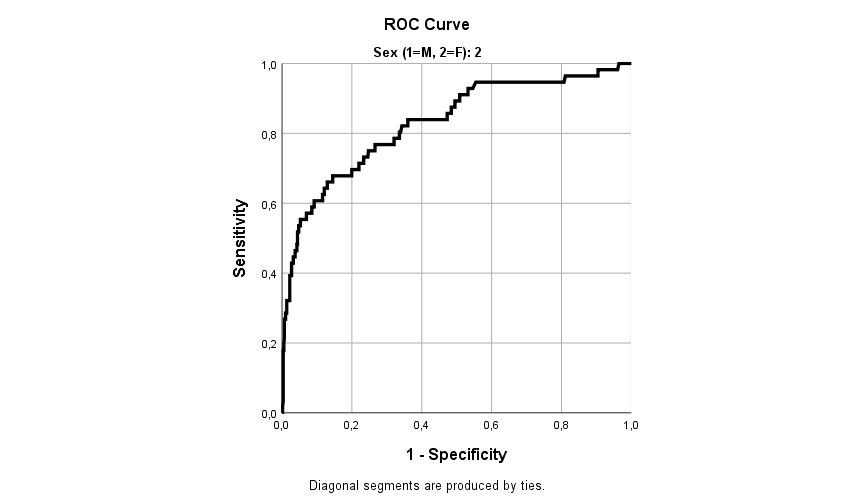


**F) Women: 6-year mortality. Optimal cut-off = 0.2149 (AUC = 0.743)**


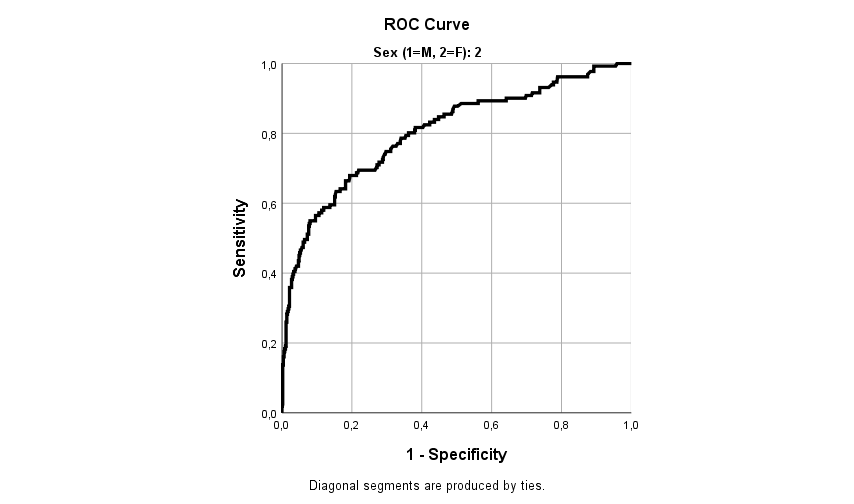


**G) Total population: 3-year CVD mortality. Optimal cut-off = 0.2014 (AUC = 0.758)**


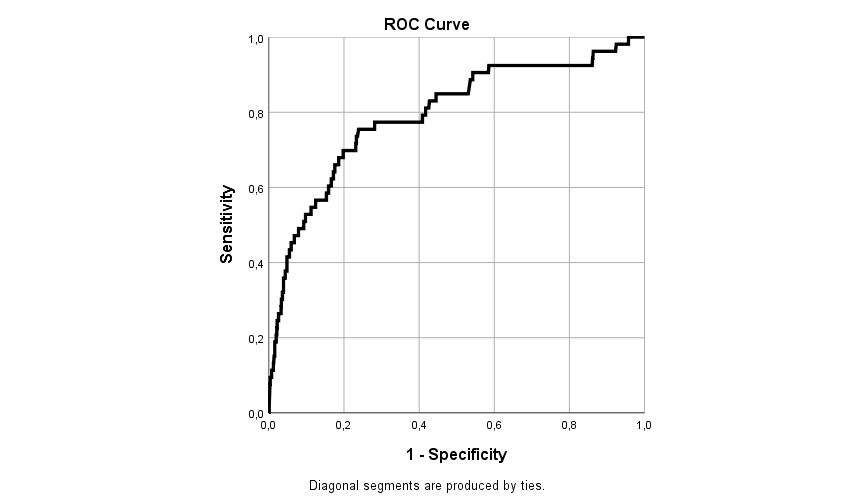


**H) Total population: 6-year CVD mortality. Optimal cut-off = 0.2014 (AUC = 0.730)**


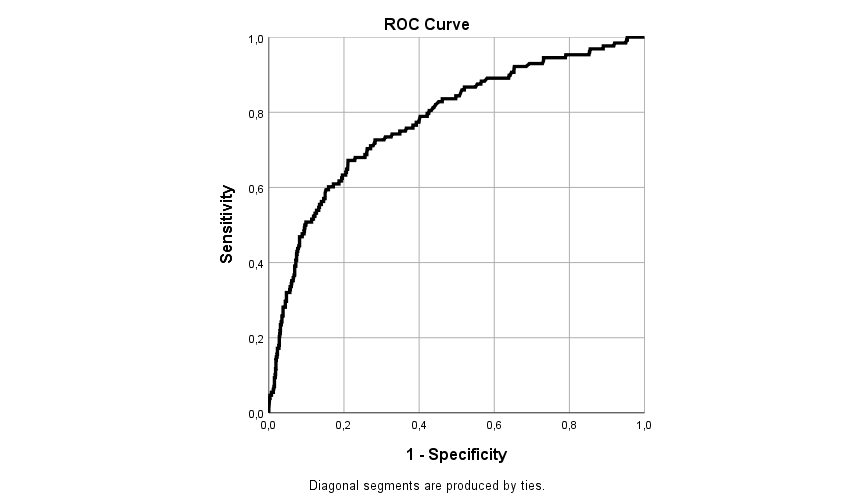


Note: AUC = Area under the ROC curve; CVD = cardiovascular disease
